# Association of Eviction With Adverse Birth Outcomes Among Women in Georgia, 2000 to 2016

**DOI:** 10.1001/jamapediatrics.2020.6550

**Published:** 2021-03-01

**Authors:** Gracie Himmelstein, Matthew Desmond

**Affiliations:** 1 Office of Population Research, Princeton University, Princeton, New Jersey; 2 Icahn School of Medicine at Mount Sinai, New York, New York; 3 Department of Sociology, Princeton University, Princeton, New Jersey

## Abstract

**Importance:**

More than 2 million families face eviction annually, a number likely to increase due to the coronavirus disease 2019 pandemic. The association of eviction with newborns’ health remains to be examined.

**Objective:**

To determine the association of eviction actions during pregnancy with birth outcomes.

**Design:**

This case-control study compared birth outcomes of infants whose mothers were evicted during gestation with those whose mothers were evicted at other times. Participants included infants born to mothers who were evicted in Georgia from January 1, 2000, to December 31, 2016. Data were analyzed from March 1 to October 4, 2020.

**Exposures:**

Eviction actions occurring during gestation.

**Main Outcomes and Measures:**

Five metrics of neonatal health included birth weight (in grams), gestational age (in weeks), and dichotomized outcomes for low birth weight (LBW) (<2500 g), prematurity (gestational age <37.0 weeks), and infant death.

**Results:**

A total of 88 862 births to 45 122 mothers (mean [SD] age, 26.26 [5.76] years) who experienced 99 517 evictions were identified during the study period, including 10 135 births to women who had an eviction action during pregnancy and 78 727 births to mothers who had experienced an eviction action when not pregnant. Compared with mothers who experienced eviction actions at other times, eviction during pregnancy was associated with lower infant birth weight (difference, −26.88 [95% CI, −39.53 to 14.24] g) and gestational age (difference, −0.09 [95% CI, −0.16 to −0.03] weeks), increased rates of LBW (0.88 [95% CI, 0.23-1.54] percentage points) and prematurity (1.14 [95% CI, 0.21-2.06] percentage points), and a nonsignificant increase in mortality (1.85 [95% CI, −0.19 to 3.89] per 1000 births). The association of eviction with birth weight was strongest in the second and third trimesters of pregnancy, with birth weight reductions of 34.74 (95% CI, −57.51 to −11.97) and 35.80 (95% CI, −52.91 to −18.69) g, respectively.

**Conclusions and Relevance:**

These findings suggest that eviction actions during pregnancy are associated with adverse birth outcomes, which have been shown to have lifelong and multigenerational consequences. Ensuring housing, social, and medical assistance to pregnant women at risk for eviction may improve infant health.

## Key Points

**Question:**

Is eviction during pregnancy associated with adverse birth outcomes, an important determinant of health across the life course?

**Findings:**

In this case-control study of 88 862 births in Georgia, eviction during pregnancy, particularly during the second and third trimester, was associated with reductions in infants’ weight and gestational age at birth compared with maternal eviction at any other time.

**Meaning:**

These findings suggest that housing, social, and medical assistance to pregnant women at risk for eviction might improve birth outcomes and health across the life course.

## Teaser

This case-control study assesses the association of eviction during pregnancy with birth outcomes among women in Georgia.

## Introduction

Nationwide, median US rent more than doubled in the last 2 decades, increasing much faster than renters’ wages.^1^ Rising housing costs have placed a growing number of US households at risk of eviction. More than 2.3 million households were served with eviction notices in 2016, and almost 900 000 people were evicted from their homes.^2^ Low-income women, Black and Hispanic women, and, especially, families with children are at high risk of eviction.^3,4^

The filing of an eviction action by a landlord causes major disruption for the tenant, often resulting in the loss of housing and possessions, forcing them into homelessness and compromising their health.^5^ The threat of eviction is acutely stressful, and those facing eviction may engage in behaviors that are particularly harmful during pregnancy, such as forgoing meals and prenatal care or engaging in physically demanding work.^6^

Eviction has been associated with numerous health harms^7,8,9,10^; 1 prior study^11^ found an ecological association between county eviction rates and birth outcomes. Other types of housing disruption have also been linked to adverse birth and childhood health outcomes,^12,13,14^ and interventions that reduce housing insecurity have health and financial benefits.^15,16,17,18,19^

Frailty at birth compromises health and economic well-being across the life course. Relative to infants with normal birth weight (2500-4000 g), those with lower birth weights have higher mortality before their first birthday and higher all-cause and cardiovascular mortality across the life course,^20^ elevated rates of failure to complete high school on time,^21^ and worse labor-market outcomes.^22,23^ Girls born at lower birth weights are more likely to eventually give birth to infants with lower birth weights.^24^ Similarly, prematurity increases the risk of immediate mortality and adverse health and socioeconomic outcomes later in life.^25,26,27^ Although low birth weight (LBW) (<2500 g) and prematurity (gestational age <37 weeks) are defined categorically using thresholds, both birth weight and gestational age show a dose-response association with adverse outcomes, even above the thresholds.^22,27^

Economic dislocation due to the coronavirus disease 2019 (COVID-19) pandemic has brought new urgency to understanding the health implications of eviction. In May 2020, one-quarter of respondents to a US Census Bureau survey reported being unable to make their most recent housing payment or had little or no confidence that they would make their next payment on time.^28^

To investigate the association between eviction and infant health, we analyzed unique data from court records and birth certificates in Georgia. Our findings highlight a potentially important and remediable contributor to multigenerational health inequalities.

## Methods

We assessed births in Georgia, a large, diverse state with longitudinally linked birth records and relatively complete eviction data. The institutional review board at Princeton University approved this research with a waiver of informed consent for the use of publicly available data, and the institutional review board at the Georgia Department of Public Health ruled this research exempt from review. This study followed the Strengthening the Reporting of Observational Studies in Epidemiology (STROBE) reporting guideline.

### Data Sources and Linkage

We obtained data on all births from January 1, 2000, to December 31, 2016, from Georgia’s Department of Health. Georgia uses a deterministic algorithm to link all births to the same mother using maternal name, date of birth, and social security number, allowing analysis of birth-certificate data for infants who share the same mother.

We obtained Georgia data on eviction actions from LexisNexis Risk Solutions, which assembles its files using a combination of automated and in-person record collection and requests to courts for bulk records.^29^ In 2016, 4.71 evictions occurred for every 100 renter homes in Georgia, more than twice the national mean.^2^

To construct the birth histories of mothers experiencing eviction actions, we matched birth and eviction records based on maternal name and residential address using a probabilistic algorithm.^30^ We identified women who gave birth while living at an address from which they had an eviction action. We then used the maternal longitudinal linkage to identify other births (at any address) to the same mother. We excluded pregnancies resulting in multiple births.

### Exposures

Georgia law allows landlords to file eviction actions against tenants for violating the lease/rental agreement or for late payment or nonpayment of rent of any duration.^31^ Once the landlord files an eviction action, the court issues a copy of the landlord’s dispossessory affidavit and a summons to the tenant. If the tenant fails to respond to the summons or the judge finds in favor of the landlord, the court may issue an eviction judgment ordering the tenant to vacate the property. Alternatively, an eviction action may end without an eviction judgment if the landlord drops the action, the court finds in favor of the tenant, or the tenant agrees in mediation to adhere to certain terms.^31^ We use the term *eviction action* to indicate any eviction filing with the court, regardless of outcome, whereas the term *eviction judgment* describes the subset of filings that result in the court finding on behalf of the plaintiff (property owner).

Our main models analyze eviction actions based on their proximity to a birth, without regard to judicial outcome because eviction actions (both the threat and experience of housing loss) impose severe duress on tenants. Analyses of eviction judgments are likely to be confounded by posttreatment conditioning, because most births after eviction judgments would not occur at the address identified in the eviction records and thus could not be matched to those records. Hence, only the subset of such mothers who had borne an older sibling while residing at the address are identifiable. For this reason, we performed sensitivity analyses excluding eviction actions that resulted in an eviction judgment.

### Modeling Strategy and Statistical Analysis

Data were analyzed from March 1 to October 4, 2020. Preliminary analyses indicated that infants born to mothers with eviction actions at any time have worse outcomes than infants born to never-evicted mothers (eFigure in the Supplement). However, mothers experiencing eviction actions differ from mothers who do not on observable characteristics such as educational level, race/ethnicity, and marital status; they likely differ in nonobservable ways as well. Accordingly, to reduce the effects of unobserved differences between evicted and nonevicted women on our analysis, our sample includes only mothers who had at least 1 eviction action during the 16-year study period. Our main analyses use variation in the timing of a birth relative to an eviction action to estimate eviction’s association with birth outcomes, considering neonates exposed to an eviction action during gestation to be cases, whereas neonates of mothers with eviction actions at any time outside of the pregnancy served as the control population.

For each mother, we estimate pooled ordinary least square models of the form: *Y_i_* = β_0_ + β_1_*Eviction_i_* + β_2_*X_i_* + β_3_*Zip code_i_* + β_4_*Year_i_* + *e*_i_, where *Y_i_* is 1 of 5 birth outcomes, including continuous measures of birth weight in grams, gestational age in weeks, and dichotomized outcomes (0, 1) for LBW (<2500 g), prematurity (gestational age <37.0 weeks), and infant death. *Eviction_i_* is 1 of 4 indicators of timing of the eviction action relative to the birth, the first, second, or third trimester as well as any time during the pregnancy, and *e_i_* is the error term. We dropped the first 9 months of 2000 birth data to avoid misclassifying births as not having an eviction action during the pregnancy. *X_i_* is a vector of maternal characteristics, including educational level, race/ethnicity, marital status, age, and parity. Because geography is an important indicator of social and environmental exposures, we include a zip code fixed effect in all models to control for all zip-code-level, place-based confounders and a year fixed effect to account for secular time trends. Reliable data on gestational age and prematurity were only available starting in 2008; analyses of those outcomes are limited to the period from 2008 to 2016.

Our main analyses examined the relationship of eviction actions during pregnancy to birth outcomes using ordinary least square models. We also estimated models for our 3 dichotomized outcome variables using logistic regression, which yielded essentially identical results and are not reported further. To examine these associations in demographic subgroups, we performed analyses stratified by race/ethnicity, marital status, educational level, and urban/rural residence.

In a sensitivity analysis, we repeated our analyses on subsamples of births. The first such analysis excluded women whose eviction actions resulted in an eviction judgment during pregnancy to minimize confounding by posttreatment conditioning. The second analysis is limited to siblings (or half-siblings), one of whom was exposed to eviction during gestation and another of whom was not. Herein, we use within-mother fixed-effect models that effectively control for all time-invariant maternal characteristics. These fixed-effect models allow the most robust inference but reduce the sample size by about 75%, creating a less representative sample and reducing statistical power. Our final 2 sensitivity analyses examine a subgroup of mothers with only a single eviction action and another subgroup evicted within Atlanta’s city limits, where 13% of Georgia’s eviction actions occur.

All analyses were performed using R, version 3.5.1 (R Foundation for Statistical Computing). Two-sided *P* < .05 indicated statistical significance.

## Results

Our sample includes 5 066 994 unique eviction actions, of which 1 992 129 (39.3%) resulted in eviction judgments. The birth records provide information on 2 239 247 singleton births to 1 524 546 mothers. After linkage of eviction actions and births, the sample includes 88 862 singleton births to 45 122 women (mean [SD] age, 26.26 [5.76] years) who experienced 99 517 eviction actions during this period. Of these, 10 135 births had an eviction action during the pregnancy (exposed group), whereas 78 727 births were to mothers who experienced an eviction action at a time when they were not pregnant (control group) (Table 1).

**Table 1.** Characteristics and Birth Outcomes of Georgia Mothers With Eviction Actions During Pregnancy vs Those With Eviction Actions at Any Other Time

Characteristic	Eviction action timing^a^	
	During pregnancy (n = 10 135)	Not during pregnancy (n = 78 727)
Maternal age, mean (SD), y	27.31 (5.35)	26.12 (5.80)
Parity		
1	1757 (17.3)	21 970 (27.9)
2	2814 (27.8)	21 838 (27.7)
3	2552 (25.2)	16 670 (21.2)
4	1528 (15.1)	9290 (11.8)
≥5	1478 (14.6)	8909 (11.3)
Missing	6 (0.1)	50 (0.1)
Race		
White	1843 (18.2)	22 487 (28.6)
American Indian or Alaska Native	17 (0.2)	84 (0.1)
Asian	43 (0.4)	601 (0.8)
Black	7896 (77.9)	53 208 (67.6)
Multiracial	242 (2.4)	1664 (2.1)
Native Hawaiian or Pacific Islander	3 (0.03)	50 (0.1)
Missing	91 (0.9)	633 (0.8)
Education		
Less than 9th grade	142 (1.4)	1878 (2.4)
9th Through 11th grade	1665 (16.4)	16 883 (21.4)
High school diploma or GED (12th grade)	4077 (40.2)	30 488 (38.7)
Some college or higher	3939 (38.9)	27 192 (34.5)
Missing	312 (3.1)	2286 (2.9)
Marital status		
Unmarried	7720 (76.2)	54 325 (69.0)
Married	2387 (23.6)	24 175 (30.7)
Missing	28 (0.3)	227 (0.3)
Infant birth weight, mean (SD), g	3105.77 (624.25)	3150.09 (600.90)
LBW (<2500 g), %	11.59	10.24
Macrosomic (>4000 g), %	4.78	5.04
Gestational age, mean (SD), wk	37.99 (2.60)	38.19 (2.41)
Premature (<37 wk), %	15.28	13.36
Infant death rate per 1000	11.35	9.50

Abbreviations: GED, General Educational Development; LBW, low birth weight.

^a^ Unless otherwise indicated, data are expressed as number (percentage) of participants.

The associations between eviction during pregnancy and birth outcomes are shown in Table 2, with coefficients for covariates presented in eTable 1 in the Supplement. Relative to births to mothers who had experienced eviction actions at any other time, eviction during gestation was associated with a decrease of 26.88 (95% CI, −39.53 to −14.24) g in infant birth weight, an increase of 0.88 (95% CI, 0.23-1.54) percentage points in the probability of being LBW, an increase of 1.85 (95% CI, −0.19 to 3.89) infant deaths per 1000, a decrease in gestational age of 0.09 (95% CI, −0.16 to −0.03) weeks, and an increase of 1.14 (95% CI, 0.21-2.06) percentage points in the rate of prematurity. The increase in infant mortality associated with eviction would be clinically important (1.85 deaths per 1000 births) but did not reach statistical significance (*P* = .08). All other decrements in infant health were statistically significant (*P* < .05). The results were similar among population subgroups (Figure). For example, decrements in birth weight were 28.50 g for Black women vs 24.80 g for White women, with significant overlap in the 95% CIs.

**Table 2.** Association of Eviction Actions During Pregnancy With Birth Outcomes, Adjusted for Other Characteristics^a^

Eviction during pregnancy	Outcome				
	Infant birth weight (95% CI), g (n = 85 438)	LBW, % of births (95% CI) (n = 85 438)	Infant deaths per 1000 (95% CI) (n = 85 438)	Gestational age (95% CI), wk (n = 42 697)	Premature, % of births (95% CI) (n = 42 697)
Any	−26.88 (−39.53 to −14.24)^b^	0.88 (0.23 to 1.54)^b^	1.85 (−0.19 to 3.89)^c^	−0.09 (−0.16 to −0.03)^b^	1.14 (0.21 to 2.06)^d^
By trimester					
First	4.34 (−21.38 to 30.07)	−0.81 (−2.14 to 0.51)	2.93 (−1.22 to 7.07)	0.05 (−0.08 to 0.18)	−0.06 (−1.93 to 1.82)
Second	−34.74 (−57.51 to −11.97)^b^	1.42 (0.25 to 2.59)^c^	2.02 (−1.65 to 5.69)	−0.12 (−0.24 to −0.01)^c^	1.41 (−0.26 to 3.09)^d^
Third	−35.80 (−52.91 to −18.69)^b^	1.31 (0.43 to 2.19)^b^	1.30 (−1.46 to 4.06)	−0.14 (−0.22 to −0.05)^b^	1.49 (0.24 to 2.73)^c^

Abbreviation: LBW, low birth weight.

^a^ Adjusted for parity (1, 2, 3, 4, or ≥5), maternal age (<19, 19-34, or ≥35 y), race (Black, White, Asian or Pacific Islander, Native American, or other), maternal educational level (<9th grade, 9th to 11th grade, high school diploma/General Educational Development, or some college or higher), and marital status. eTables 1 and 2 in the Supplement provide estimates of these covariates. Ordinary least squares models use with fixed effects for zip code and year.

^b^ *P* < .01.

^c^ *P* < .05.

^d^ *P* < .10.

**Figure.**
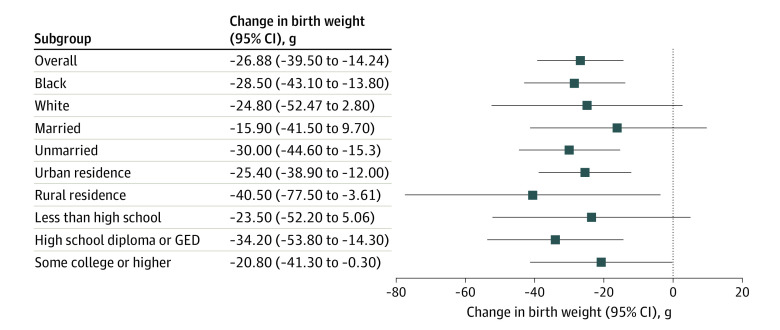
Estimates of Association of Eviction With Infant Birth Weight Overall and by Subgroup Data are from court records and birth certificates in Georgia from 2000 to 2016. GED indicates General Educational Development.

Table 2 also displays outcomes according to the trimester of pregnancy during which the eviction action occurred with the coefficients for covariates presented in eTable 2 in the Supplement. Although the sociodemographic characteristics of women evicted in each trimester of pregnancy were similar (eTable 3 in the Supplement), the association of eviction with their birth outcomes differed. Eviction actions had the strongest association with birth outcomes during trimesters 2 and 3, when most fetal weight gain occurs.^32^ Eviction actions during the second and third trimesters were associated with birth weight reductions of 34.74 (95% CI, −57.51 to −11.97) and 35.80 (95% CI, −52.91 to −18.69) g, respectively, a significant increase of 1.42 (95% CI, 0.25-2.59) and 1.31 (95% CI, 0.43-2.19) percentage points, respectively, in the probability of LBW, and declines in gestational age of 0.12 (95% CI, −0.24 to −0.01) to 0.14 (95% CI, −0.22 to −0.05) weeks, respectively.

Sensitivity analyses limited to the subgroup of mothers whose eviction action did not result in eviction judgments (to minimize posttreatment conditioning) yielded similar results to our main analyses (eTable 4 in the Supplement). Further sensitivity analyses of the subgroup of women who had an eviction action during one pregnancy and not during another pregnancy (using models with within-mother fixed effects) yielded estimates numerically consistent with our main analyses, although only the estimate for the association between an eviction action during pregnancy and mean infant birth weight remained statistically significant at the *P* < .05 level (−18.87; 95% CI, −33.59 to −4.15) (eTable 5 in the Supplement). Sensitivity analyses of additional subsamples—births to women with just 1 eviction action (eTable 6 in the Supplement) and births in Atlanta (eTable 7 in the Supplement)—also yielded numerically similar findings.

## Discussion

Eviction actions during pregnancy, as opposed to eviction actions at any other time, were associated with worse birth outcomes, including reduced birth weight, shorter gestation, increased probability of being classified as LBW or premature, and a trend toward increased infant mortality. Analyses using within-mother fixed-effects models, which used other births to the same mother as controls, yielded similar results, supporting the conclusion that eviction per se, rather than factors intrinsic to evicted mothers, explain the observed birth-weight reductions. The size of the decrements in birth weight were similar in magnitude to the estimated birth-weight gains from nutritional support programs for pregnant women, such as the Supplemental Nutritional Assistance Program and the Special Supplemental Nutrition Program for Women, Infants, and Children.^33,34^ Eviction disproportionately affects Black mothers, who are represented in our sample at a rate of 2.5 to 1.0, compared with White mothers. Given the lifelong and intergenerational consequences of frailty at birth, failure to prevent eviction could compound the population-health harms of the COVID-19 pandemic.

Eviction may affect pregnancy outcomes through several mechanisms, acting alone or in concert, including changes in maternal health behaviors or disruptions in prenatal care. Evictions may also adversely affect birth outcomes by inducing acute stress, precipitating moves into homeless shelters or substandard housing, or forcing mothers to double up with others, which may increase exposure to infectious agents, environmental toxins, sexual coercion, or violence.^35^ Maternal stress may harm a fetus directly, via alterations in maternal neuroendocrine responses, or indirectly, via effects on maternal health (eg, hypertension).^36^

Our findings that eviction actions during the second and third trimester are most strongly associated with decrements in infant health are consistent with studies of fetal exposure to other adverse conditions. Landmark studies of infants born to mothers who survived the 1944-1945 Dutch Hunger Winter^37,38^ found that famine exposure during the second and third trimesters reduced birth weight, whereas famine exposure in the first trimester did not. Similarly, studies of infants gestating during the 9/11 terrorist attacks and of Israeli infants exposed to increased rocket attacks^39,40,41^ found maternal stress during the second and third trimester of pregnancy especially detrimental. Other studies^42,43,44^ indicate that exposure to environmental toxins during the second and third trimesters is particularly harmful to infant birth weight.

Several studies have documented associations between other types of housing disruption and adverse birth outcomes. A survey of pregnant women (in their second trimester) at community hospitals in New York City^12^ found that housing instability (defined as ≥2 moves in the previous year) was associated with lower birth weights. Among 200 homeless women who had given birth in the previous 3 years, birth outcomes and racial disparities in birth outcomes were worse than in the general population, and severity of homelessness (ie, percentage of life spent homeless and number of times homeless) was monotonically related to adverse birth outcomes.^45^ A survey of 9995 mothers in emergency departments and clinics in 5 cities found that homelessness during pregnancy (but not after delivery) was associated with increased odds of giving birth to an LBW or preterm infant.^13^ Among women initially residing in public housing, those forced to move because of demolition were more likely to deliver a preterm or LBW infant.^46^

Our study adds a population-based perspective on the association between housing dynamics and infant health. In addition, unlike poverty and homelessness, eviction offers a discrete and tractable target for intervention. Policies that improve housing affordability, or even more modest reforms like ensuring legal assistance in eviction court, might reduce the likelihood of eviction.^47^

Hospital systems may promote housing stability among their patients by providing rent support, investing in affordable housing, or creating partnerships to integrate legal resources into medical care.^48,49^ Clinicians may contribute to these efforts by integrating screening questionnaires into their practice to help facilitate patient access to such resources.^49,50^

### Limitations

Our study has several limitations. To minimize unobserved confounding, our control group was constructed from births to women who experienced eviction actions, but not during the gestation. Although these controls were not exposed to the acute effects of eviction during gestation, some might have been minimally exposed if they experienced the long-term sequelae of eviction, thereby biasing our estimates toward the null.

We were more likely to identify births and evictions occurring close to each other in time, owing to our use of addresses to link births to evictions. A woman evicted at a different address than that listed on the birth certificate (the likely scenario after an eviction judgment early in the pregnancy) was unlikely to be matched with a birth record. Our linkage would not identify pregnancies in mothers who experienced the most severe housing instability (eg, sheriff-implemented eviction, with possessions removed from the home), leading us to undercount evictions during gestation and (again) likely biasing our findings toward the null.

As in any observational analysis of exposures such as eviction that cannot be randomized, causality cannot be proven. We use variation in the timing of eviction actions relative to births and maternal fixed-effect models to account for unobserved confounders that might influence the association between eviction actions and birth outcomes. These approaches cannot account for time-varying factors operating in the very short term (ie, with onset during the later trimesters of pregnancy) that caused both adverse birth outcomes and eviction actions.

However, rather than being regularly precipitated by a shock such as job loss or a medical emergency, most evictions reflect the chronic precarity of renters and the scant legal protections available to them. Most poor tenants nationwide spend at least half of their income on housing, with 1 in 4 spending more than 70%.^51^ An analysis of millions of eviction records from 12 states revealed that 1 in 3 eviction actions were for less than 1 month’s worth of rent.^52^

## Conclusions

We find strong evidence that maternal eviction is associated with clinically meaningful decrements in virtually all available indicators of newborn health. Policies to protect tenants from eviction might improve child health.

## Notes

Supplement. **eFigure.** Birth Weight Distributions of Infants Born to Georgia Mothers With and Without Eviction Actions at Any Time, 2000-2016 **eTable 1.** Association of Eviction Actions During Pregnancy With Birth Outcomes, Adjusted for Other Characteristics, Including Coefficients for All Covariates **eTable 2.** Association of Eviction Actions During Pregnancy With Birth Outcomes According to Trimester of Pregnancy, Adjusted for Other Characteristics, Including Coefficients for All Covariates **eTable 3.** Characteristics and Birth Outcomes of Georgia Mothers With Eviction Actions During Pregnancy by Trimester **eTable 4.** Association of Eviction Actions Not Resulting in Eviction Judgment During Pregnancy With Birth Outcomes, Adjusted for Other Characteristics **eTable 5.** Association of Eviction Actions During Pregnancy With Birth Outcomes, Including Mother Fixed Effects, Adjusted for Other Characteristics **eTable 6.** Association of Eviction Actions During Pregnancy With Birth Outcomes Among Mothers With Only 1 Eviction Action, Adjusted for Other Characteristics **eTable 7.** Association of Eviction Actions During Pregnancy With Birth Outcomes in Atlanta, Georgia, Adjusted for Other Characteristics
