# Supplementary material for: Association of Eviction With Adverse Birth Outcomes Among Women in Georgia, 2000 to 2016
Source: JAMA Pediatr. 2021 Mar 1;175(5):1–7. doi: 10.1001/jamapediatrics.2020.6550 (PMC7922232; doi:10.1001/jamapediatrics.2020.6550)
Supplement: Supplement. — eFigure. Birth Weight Distributions of Infants Born to Georgia Mothers With and Without Eviction Actions at Any Time, 2000-2016 eTable 1. Association of Eviction Actions During Pregnancy With Birth Outcomes, Adjusted for Other Characteristics, Including Coefficients for All Covariates eTable 2. Association of Eviction Actions During Pregnancy With Birth Outcomes According to Trimester of Pregnancy, Adjusted for Other Characteristics, Including Coefficients for All Covariates eTable 3. Characteristics and Birth Outcomes of Georgia Mothers With Eviction Actions During Pregnancy by Trimester eTable 4. Association of Eviction Actions Not Resulting in Eviction Judgment During Pregnancy With Birth Outcomes, Adjusted for Other Characteristics eTable 5. Association of Eviction Actions During Pregnancy With Birth Outcomes, Including Mother Fixed Effects, Adjusted for Other Characteristics eTable 6. Association of Eviction Actions During Pregnancy With Birth Outcomes Among Mothers With Only 1 Eviction Action, Adjusted for Other Characteristics eTable 7. Association of Eviction Actions During Pregnancy With Birth Outcomes in Atlanta, Georgia, Adjusted for Other Characteristics [file jamapediatr-e206550-s001.pdf]

Supplementary Online Content

Himmelstein G, Desmond M. Association of eviction with adverse birth outcomes among women in Georgia, 2000 to 2016. *JAMA Pediatr*. Published online March 1, 2021. doi:10.1001/jamapediatrics.2020.6550

eFigure. Birth Weight Distributions of Infants Born to Georgia Mothers With and Without Eviction Actions at Any Time, 2000-2016

eTable 1. Association of Eviction Actions During Pregnancy With Birth Outcomes, Adjusted for Other Characteristics, Including Coefficients for All Covariates

eTable 2. Association of Eviction Actions During Pregnancy With Birth Outcomes According to Trimester of Pregnancy, Adjusted for Other Characteristics, Including Coefficients for All Covariates

eTable 3. Characteristics and Birth Outcomes of Georgia Mothers With Eviction Actions During Pregnancy by Trimester

eTable 4. Association of Eviction Actions Not Resulting in Eviction Judgment During Pregnancy With Birth Outcomes, Adjusted for Other Characteristics

eTable 5. Association of Eviction Actions During Pregnancy With Birth Outcomes, Including Mother Fixed Effects, Adjusted for Other Characteristics

eTable 6. Association of Eviction Actions During Pregnancy With Birth Outcomes Among Mothers With Only 1 Eviction Action, Adjusted for Other Characteristics

eTable 7. Association of Eviction Actions During Pregnancy With Birth Outcomes in Atlanta, Georgia, Adjusted for Other Characteristics

This supplementary material has been provided by the authors to give readers additional information about their work.

©2021 Himmelstein G et al. *JAMA Pediatrics*.

eFigure. Birth Weight Distributions of Infants Born to Georgia Mothers With and Without Eviction Actions at Any Time, 2000-2016

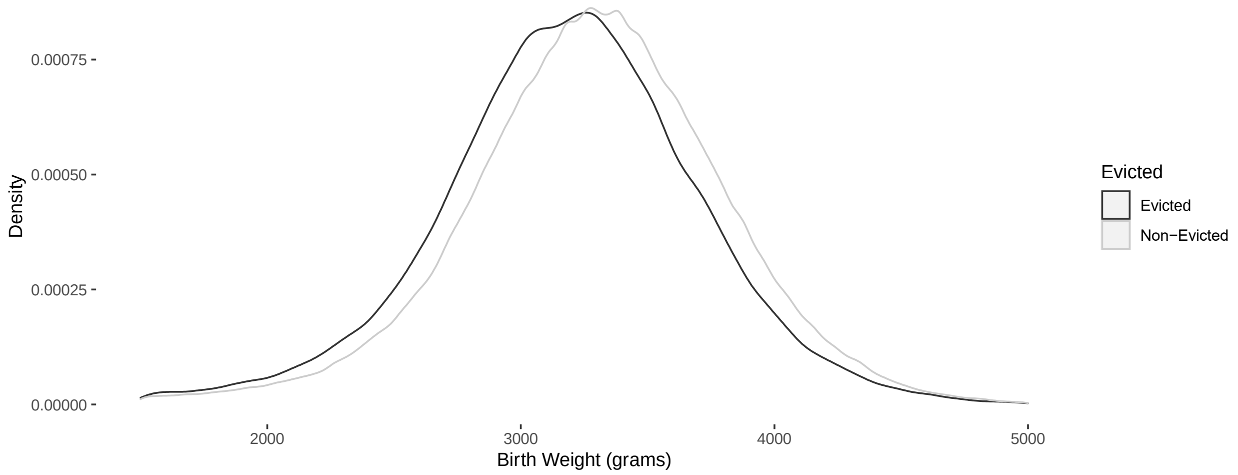

©2021 Himmelstein G et al. *JAMA Pediatrics*.

eTable 1. Association of Eviction Actions During Pregnancy With Birth Outcomes, Adjusted for Other Characteristics, Including Coefficients for All Covariates

	**Infant Birth Weight, Grams** N = 85,438 (95% CI)	**Low Birth Weight, Percent of Births** N = 85,438 (95% CI)	**Infant Deaths per Thousand** N = 85,438 (95% CI)	**Gestational Age, Weeks** N= 42,697 (95% CI)	**Premature, Percent of Births** N = 42,697 (95% CI)
**Eviction Action During Pregnancy**	**-26.88*****<br>(-39.53, -14.24)	**0.88*****<br>(0.23, 1.54)	**1.85***<br>(-0.19, 3.89)	**-0.09*****<br>(-0.16, -0.03)	**1.14****<br>(0.21, 2.06)
**Parity**					
Parity 1	Reference	Reference	Reference	Reference	Reference
Parity 2	61.61***<br>(50.54, 72.67)	-2.55***<br>(-3.12, -1.98)	-1.78*<br>(-3.57, 0.00)	-0.11***<br>(-0.17, -0.04)	-0.01<br>(-0.94, 0.92)
Parity 3	72.90***<br>(60.87, 84.92)	-2.53***<br>(-3.15, -1.91)	-0.94<br>(-2.88, 0.99)	-0.16***<br>(-0.23, -0.09)	0.74<br>(-0.23, 1.71)
Parity 4	51.11***<br>(36.73, 65.50)	-1.60***<br>(-2.34, -0.86)	0.48<br>(-1.84, 2.79)	-0.27***<br>(-0.35, -0.19)	2.08***<br>(0.97, 3.20)
Parity 5+	48.65***<br>(33.64, 63.67)	-1.06***<br>(-1.83, -0.28)	0.31<br>(-2.11, 2.73)	-0.35***<br>(-0.42, -0.27)	3.71***<br>(2.59, 4.83)
**Maternal Age**					
< 19 years	Reference	Reference	Reference	Reference	Reference
19-34 years	1.18<br>(-17.01, 19.37)	0.82*<br>(-0.12, 1.75)	1.66<br>(-1.27, 4.59)	-0.11*<br>(-0.24, 0.02)	1.77*<br>(-0.03, 3.57)
35+ years	-24.80**<br>(-47.85, -1.76)	3.96***<br>(2.78, 5.15)	1.54<br>(-2.18, 5.25)	-0.35***<br>(-0.49, -0.20)	5.57***<br>(3.50, 7.64)
**Maternal Race**					
White	Reference	Reference	Reference	Reference	Reference
Black or African-American	-185.48***<br>(-196.83, -174.12)	4.39***<br>(3.81, 4.98)	3.04***<br>(1.21, 4.87)	-0.30***<br>(-0.37, -0.24)	3.16***<br>(2.25, 4.08)
**Maternal Education**					
< 9th grade	Reference	Reference	Reference	Reference	Reference
9th through 11th Grade	-43.53***<br>(-71.21, -15.85)	1.12<br>(-0.31, 2.55)	-1.17<br>(-5.64, 3.29)	-0.24**<br>(-0.42, -0.05)	3.40**<br>(0.78, 6.02)
High School Diploma/GED	4.57<br>(-22.77, 31.91)	-0.15<br>(-1.56, 1.25)	-2.15<br>(-6.56, 2.26)	-0.20**<br>(-0.38, -0.02)	2.32*<br>(-0.25, 4.88)

©2021 Himmelstein G et al. *JAMA Pediatrics*.

Some College or Higher	35.20** (7.47, 62.93)	-1.10 (-2.52, 0.33)	-4.30* (-8.77, 0.17)	-0.16* (-0.34, 0.02)	2.00 (-0.58, 4.59)
**Marital status**					
Unmarried	Reference	Reference	Reference	Reference	Reference
Married	55.32*** (45.43, 65.22)	-1.28*** (-1.79, -0.77)	-0.78 (-2.37, 0.81)	0.02 (-0.04, 0.08)	-0.74* (-1.53, 0.05)

*p<0.1; **p<0.05; ***p<0.01
Multivariate ordinary least squares models with zip code and year fixed effects
GED= General Education Diploma

©2021 Himmelstein G et al. *JAMA Pediatrics*.

eTable 2. Association of Eviction Actions During Pregnancy With Birth Outcomes According to Trimester of Pregnancy, Adjusted for Other Characteristics, Including Coefficients for All Covariates

	**Infant Birth Weight, Grams** N = 85,438 (95% CI)	**Low Birth Weight, Percent of Births** N = 85,438 (95% CI)	**Infant Deaths per Thousand** N = 85,438 (95% CI)	**Gestational Age, Weeks** N= 42,697 (95% CI)	**Premature, Percent of Births** N = 42,697 (95% CI)
**Trimester During Which Eviction Occurred**					
1st Trimester of Pregnancy	4.34 (-21.38, 30.07)	-0.81 (-2.14, 0.51)	2.93 (-1.22, 7.07)	0.05 (-0.08, 0.18)	-0.06 (-1.93, 1.82)
2nd Trimester of Pregnancy	-34.74*** (-57.51, -11.97)	1.42** (0.25, 2.59)	2.02 (-1.65, 5.69)	-0.12** (-0.24, -0.01)	1.41* (-0.26, 3.09)
3rd Trimester of Pregnancy	-35.80*** (-52.91, -18.69)	1.31*** (0.43, 2.19)	1.30 (-1.46, 4.06)	-0.14*** (-0.22, -0.05)	1.49** (0.24, 2.73)
**Parity**					
Parity 1	Reference	Reference	Reference	Reference	Reference
Parity 2	61.43*** (50.36, 72.49)	-2.54*** (-3.11, -1.97)	-1.79** (-3.58, -0.01)	-0.11*** (-0.17, -0.04)	0.00 (-0.93, 0.93)
Parity 3	72.61*** (60.59, 84.64)	-2.51*** (-3.13, -1.89)	-0.96 (-2.90, 0.98)	-0.16*** (-0.23, -0.09)	0.76 (-0.21, 1.73)
Parity 4	50.84*** (36.45, 65.22)	-1.58*** (-2.32, -0.84)	0.46 (-1.85, 2.78)	-0.27*** (-0.35, -0.20)	2.10*** (0.98, 3.21)
Parity 5+	48.52*** (33.50, 63.54)	-1.05*** (-1.82, -0.28)	0.31 (-2.12, 2.73)	-0.35*** (-0.43, -0.27)	3.72*** (2.59, 4.84)
**Maternal Age**					
Age < 19	Reference	Reference	Reference	Reference	Reference
Age 19-34	1.20 (-16.99, 19.38)	0.81* (-0.12, 1.75)	1.66 (-1.27, 4.60)	-0.11* (-0.24, 0.02)	1.77* (-0.03, 3.57)
Age 35+	-24.73** (-47.77, -1.68)	3.96*** (2.77, 5.15)	1.54 (-2.18, 5.25)	-0.35*** (-0.49, -0.20)	5.57*** (3.49, 7.64)
**Maternal Race**					
White	Reference	Reference	Reference	Reference	Reference

©2021 Himmelstein G et al. *JAMA Pediatrics*.

Black or African-American	-185.34*** (-196.70, -173.99)	4.39*** (3.80, 4.97)	3.05*** (1.21, 4.88)	-0.30*** (-0.37, -0.24)	3.16*** (2.24, 4.07)
**Maternal Education**					
< 9th Grade	Reference	Reference	Reference	Reference	Reference
9th through 11th Grade	-43.66*** (-71.34, -15.98)	1.13 (-0.30, 2.55)	-1.18 (-5.64, 3.28)	-0.24** (-0.42, -0.05)	3.41** (0.79, 6.03)
High School Diploma or GED	4.50 (-22.84, 31.84)	-0.15 (-1.56, 1.26)	-2.15 (-6.56, 2.25)	-0.20** (-0.38, -0.02)	2.32* (-0.25, 4.89)
Some College or Higher	35.11** (7.38, 62.84)	-1.09 (-2.52, 0.34)	-4.31* (-8.78, 0.16)	-0.16* (-0.34, 0.02)	2.01 (-0.58, 4.59)
**Marital Status**					
Unmarried	Reference	Reference	Reference	Reference	Reference
Married	55.38*** (45.49, 65.27)	-1.28*** (-1.79, -0.77)	-0.000.78 (-2.37, 0.82)	0.02 (-0.04, 0.08)	-0.74* (-1.53, 0.05)

*p<0.1; **p<0.05; ***p<0.01
Multivariate ordinary least squares models with zip code and year fixed effects
GED= General Education Diploma

©2021 Himmelstein G et al. *JAMA Pediatrics*.

eTable 3. Characteristics and Birth Outcomes of Georgia Mothers With Eviction Actions During Pregnancy by Trimester

	**Eviction Action During First Trimester of Pregnancy**	**Eviction Action During Second Trimester of Pregnancy**	**Eviction Action During Third Trimester of Pregnancy**
	N=2,199	N=2,802	N=5,134
**Mean Maternal Age (SD)**	27.18 (5.29)	27.41 (5.41)	27.31 (5.36)
**Race (%)**			
White	445 (20.2)	535 (19.1)	863 (16.8)
American Indian/Alaska Native	2 (0.1)	6 (0.2)	9 (0.2)
Asian	10 (0.5)	15 (0.5)	18 (0.4)
Black or African-American	1669 (75.9)	2152 (76.8)	4075 (79.4)
Multiracial	48 (2.2)	69 (2.5)	125 (2.4)
Native Hawaiian/Pacific Islander	0 (0.0)	1 (0.0)	2 (0.0)
Unknown	25 (1.1)	24 (0.9)	42 (0.8)
**Education (%)**			
Less than 9th Grade	27 (1.2)	38 (1.4)	77 (1.5)
9th through 11th Grade	402 (18.3)	460 (16.4)	803 (15.6)
High School Diploma or GED	875 (39.8)	1138 (40.6)	2064 (40.2)
Some College or Higher	809 (36.8)	1081 (38.6)	2049 (39.9)
Missing	86 (3.9)	85 (3.0)	141 (2.7)
**Marital Status (%)**			
Unmarried	1693 (77.0)	2127 (75.9)	3900 (76.0)
Married	501 (22.8)	669 (23.9)	1217 (23.7)
Missing	5 (0.2)	6 (0.2)	17 (0.3)

©2021 Himmelstein G et al. *JAMA Pediatrics*.

eTable 4. Association of Eviction Actions Not Resulting in Eviction Judgment During Pregnancy With Birth Outcomes, Adjusted for Other Characteristics

	**Infant Birth Weight, Grams N = 57,256 (95% CI)**	**Low Birth Weight, Percent of Births N = 57,256 (95% CI)**	**Infant Deaths per Thousand N = 57,256 (95% CI)**	**Gestational Age, Weeks N= 28,864 (95% CI)**	**Premature, Percent of Births N = 28,864 (95% CI)**
**Eviction Action During Pregnancy**	**-29.25***** (-43.69, -14.81)	**1.10***** (0.35, 1.85)	**1.72** (-0.62, 4.06)	**-0.11***** (-0.19, -0.03)	**1.47***** (0.40, 2.54)
**Parity**					
Parity 1	Reference	Reference	Reference	Reference	Reference
Parity 2	60.29*** (46.55, 74.03)	-2.76*** (-3.48, -2.05)	-1.38 (-3.61, 0.85)	-0.07* (-0.16, 0.01)	-0.46 (-1.60, 0.69)
Parity 3	72.91*** (58.05, 87.77)	-2.64*** (-3.41, -1.87)	-1.25 (-3.66, 1.16)	-0.13*** (-0.22, -0.05)	0.18 (-1.02, 1.37)
Parity 4	42.98*** (25.32, 60.64)	-1.52*** (-2.44, -0.60)	1.62 (-1.24, 4.48)	-0.28*** (-0.38, -0.19)	2.13*** (0.76, 3.50)
Parity 5+	42.22*** (23.97, 60.48)	-0.99** (-1.94, -0.04)	0.25 (-2.71, 3.21)	-0.32*** (-0.42, -0.23)	3.33*** (1.96, 4.71)
**Maternal Age**					
Age <19	Reference	Reference	Reference	Reference	Reference
Age 19-34	2.23 (-20.32, 24.78)	0.50 (-0.67, 1.67)	2.26 (-1.39, 5.92)	-0.12 (-0.28, 0.04)	1.72 (-0.56, 4.00)
Age 35+	-25.55* (-54.22, 3.12)	3.73*** (2.24, 5.22)	2.54 (-2.11, 7.18)	-0.36*** (-0.54, -0.17)	5.39*** (2.79, 8.00)
**Maternal race**					
White	Reference	Reference	Reference	Reference	Reference
Black or African-American	-180.74*** (-195.45, -166.03)	4.41*** (3.65, 5.17)	4.04*** (1.65, 6.42)	-0.25*** (-0.34, -0.17)	2.45*** (1.27, 3.62)
**Maternal education**					
< 9th Grade	Reference	Reference	Reference	Reference	Reference
9th through 11th Grade	-36.88** (-71.87, -1.88)	0.62 (-1.20, 2.44)	-0.93 (-6.60, 4.74)	-0.16 (-0.40, 0.07)	1.99 (-1.33, 5.31)
High School Diploma or GED	8.43 (-26.16, 43.02)	-0.70 (-2.50, 1.10)	-2.40 (-8.01, 3.21)	-0.10 (-0.33, 0.13)	0.73 (-2.52, 3.99)
Some College or Higher	39.54** (4.42, 74.65)	-1.81* (-3.63, 0.02)	-4.51 (-10.20, 1.18)	-0.08 (-0.31, 0.15)	0.79 (-2.49, 4.06)

©2021 Himmelstein G et al. *JAMA Pediatrics*.

**Marital Status**					
Unmarried	Reference	Reference	Reference	Reference	Reference
Married	52.91*** (40.46, 65.36)	-1.14*** (-1.78, -0.49)	-0.67 (-2.69, 1.35)	0.01 (-0.06, 0.08)	-0.61 (-1.60, 0.38)

*p<0.1; **p<0.05; ***p<0.01
Note: Ordinary least squares models with zip code and year fixed effects
GED= General Education Diploma

©2021 Himmelstein G et al. *JAMA Pediatrics*.

eTable 5. Association of Eviction Actions During Pregnancy With Birth Outcomes, Including Mother Fixed Effects, Adjusted for Other Characteristics

	**Infant Birth Weight, Grams N = 85,438 (95% CI)**	**Low Birth Weight, Percent of Births N = 85,438 (95% CI)**	**Infant Deaths per Thousand N = 85,438 (95% CI)**	**Gestational Age, Weeks N= 42,697 (95% CI)**	**Premature, Percent of Births N = 42,697 (95% CI)**
**Eviction Action During Pregnancy**	**-18.87**** (-33.59, -4.15)	**0.56** (-0.32, 1.43)	**1.10** (-2.11, 4.31)	**-0.04** (-0.14, 0.05)	**0.61** (-0.82, 2.04)
**Parity**					
Parity 1	Reference	Reference	Reference	Reference	Reference
Parity 2	59.15*** (46.70, 71.60)	-2.65*** (-3.39, -1.90)	-1.91 (-4.63, 0.80)	-0.15*** (-0.24, -0.06)	0.74 (-0.64, 2.12)
Parity 3	62.49*** (45.50, 79.48)	-2.55*** (-3.57, -1.54)	-0.09 (-3.79, 3.61)	-0.18*** (-0.31, -0.06)	1.28 (-0.58, 3.13)
Parity 4	47.69*** (25.71, 69.66)	-1.91*** (-3.23, -0.60)	-0.66 (-5.45, 4.13)	-0.32*** (-0.48, -0.16)	3.98*** (1.61, 6.34)
Parity 5+	36.59*** (9.22, 63.95)	-1.70** (-3.33, -0.06)	0.97 (-5.00, 6.93)	-0.45*** (-0.64, -0.25)	5.40*** (2.47, 8.33)
**Maternal Age**					
Age < 19	Reference	Reference	Reference	Reference	Reference
Age 19-34	-21.11** (-40.52, -1.70)	0.75 (-0.41, 1.91)	1.19 (-3.04, 5.43)	-0.08 (-0.25, 0.08)	2.50** (0.07, 4.94)
Age 35+	-46.54*** (-79.29, - 13.79)	2.30** (0.34, 04.25)	-3.14 (-10.28, 04.00)	-0.07 (-0.32, 0.19)	4.00** (0.20, 0.07.80)

*p<0.1; **p<0.05; ***p<0.01

Note: Ordinary least squares models with mother, zip code and year fixed effects

©2021 Himmelstein G et al. *JAMA Pediatrics*.

eTable 6. Association of Eviction Actions During Pregnancy With Birth Outcomes Among Mothers With Only 1 Eviction Action, Adjusted for Other Characteristics

	**Infant Birth Weight, Grams** N = 40,858 (95% CI)	**Low Birth Weight, Percent of Births** N = 40,858 (95% CI)	**Infant Deaths per Thousand** N = 40,858 (95% CI)	**Gestational Age, Weeks** N= 20,192 (95% CI)	**Premature, Percent of Births** N = 20,192 (95% CI)
**Eviction Action During Pregnancy**	-44.95*** (-68.23, -21.68)	1.55** (0.36, 2.73)	5.56*** (1.92, 9.20)	-0.16*** (-0.27, -0.04)	1.78** (0.11, 3.44)
**Parity**					
Parity 1	Reference	Reference	Reference	Reference	Reference
Parity 2	58.15*** (42.55, 73.76)	-2.47*** (-3.27, -1.68)	-0.81 (-3.25, 1.63)	-0.12** (-0.21, -0.03)	-0.12 (-1.45, 1.20)
Parity 3	64.43*** (47.28, 81.59)	-2.13*** (-3.00, -1.25)	-0.12 (-2.81, 2.56)	-0.21*** (-0.31, -0.12)	1.12 (-0.27, 2.52)
Parity 4	46.70*** (25.83, 67.56)	-2.05*** (-3.11, -0.99)	0.44 (-2.83, 3.71)	-0.26*** (-0.37, -0.15)	1.63** (0.02, 3.24)
Parity 5+	45.97*** (23.44, 68.51)	-1.25** (-2.40, -0.10)	1.31 (-2.22, 4.83)	-0.36*** (-0.48, -0.25)	4.13*** (2.45, 5.81)
**Maternal Age**					
Age <19	Reference	Reference	Reference	Reference	Reference
Age 19-34	-0.60 (-26.35, 25.15)	1.30* (-0.02, 2.61)	1.26 (-2.77, 5.29)	-0.07 (-0.24, 0.11)	1.96 (-0.55, 4.47)
Age 35+	-13.83 (-46.70, 19.03)	3.69*** (2.01, 5.36)	0.30 (-4.84, 5.45)	-0.19* (-0.40, 0.01)	4.08*** (1.14, 7.02)
**Maternal Race**					
White	Reference	Reference	Reference	Reference	Reference
Black or African-American	-182.60*** (-198.48, -166.72)	4.49*** (3.68, 5.30)	2.41* (-0.07, 4.90)	-0.27*** (-0.36, -0.18)	2.40*** (1.12, 3.67)
**Maternal Education**					
<9th Grade	Reference	Reference	Reference	Reference	Reference

©2021 Himmelstein G et al. *JAMA Pediatrics*.

9th through 11th Grade	-61.22*** (-99.57, -22.88)	1.69* (-0.26, 3.65)	-0.77 (-6.77, 5.23)	-0.29** (-0.55, -0.04)	3.84** (0.17, 7.51)
High School Diploma or GED	-8.32 (-46.21, 29.56)	0.04 (-1.89, 1.97)	-2.63 (-8.56, 3.30)	-0.24* (-0.49, 0.01)	2.79 (-0.81, 6.40)
Some College or Higher	25.32 (-13.23, 63.87)	-0.98 (-2.94, 0.99)	-3.83 (-9.87, 2.20)	-0.17 (-0.42, 0.08)	2.23 (-1.41, 5.87)
**Marital Status**					
Unmarried	Reference	Reference	Reference	Reference	Reference
Married	56.05*** (42.15, 69.94)	-1.19*** (-1.89, -0.48)	-0.34 (-2.52, 1.83)	-0.02 (-0.10, 0.06)	-0.36 (-1.48, 0.76)

*p<0.1; **p<0.05; ***p<0.01
Notes: Ordinary least squares models with zip code and year fixed effects
GED= General Education Diploma

©2021 Himmelstein G et al. *JAMA Pediatrics*.

eTable 7. Association of Eviction Actions During Pregnancy With Birth Outcomes in Atlanta, Georgia, Adjusted for Other Characteristics

	**Infant Birth Weight, Grams** N = 14,332 (95% CI)	**Low Birth Weight, Percent of Births** N = 14,332 (95% CI)	**Infant Deaths per Thousand** N = 14,332 (95% CI)	**Gestational Age, Weeks** N= 6,855 (95% CI)	**Premature, Percent of Births** N = 6,855 (95% CI)
**Eviction Action During Pregnancy**	**-38.97***** (-68.44, -9.49)	**2.46***** (0.89, 4.03)	**0.37** (-4.32, 5.05)	**-0.18**** (-0.35, -0.02)	**1.61** (-0.70, 3.92)
**Parity**					
Parity 1	Reference	Reference	Reference	Reference	Reference
Parity 2	77.29*** (49.21, 105.37)	-3.85*** (-5.34, -2.35)	-5.91*** (-10.37, -1.45)	-0.00 (-0.17, 0.17)	-0.89 (-3.32, 1.55)
Parity 3	108.02*** (77.24, 138.81)	-3.58*** (-5.22, -1.93)	-3.71 (-8.60, 1.18)	0.01 (-0.17, 0.19)	-0.71 (-3.28, 1.86)
Parity 4	53.47*** (17.86, 89.08)	-1.57 (-3.47, 0.33)	-1.49 (-7.15, 4.17)	-0.06 (-0.27, 0.15)	0.47 (-2.43, 3.37)
Parity 5+	40.14** (5.43, 74.85)	-0.57 (-2.43, 1.28)	-1.53 (-7.05, 3.98)	-0.26*** (-0.46, -0.07)	5.15*** (2.41, 7.89)
**Maternal Age**					
Age < 19	Reference	Reference	Reference	Reference	Reference
Age 19-34	9.03 (-34.12, 52.19)	0.50 (-1.81, 2.80)	1.31 (-5.55, 8.16)	0.03 (-0.30, 0.36)	0.44 (-4.18, 5.07)
Age 35+	13.20 (-43.87, 70.28)	2.91* (-0.14, 5.95)	-1.77 (-10.84, 7.30)	-0.05 (-0.43, 0.33)	2.04 (-3.33, 7.41)
**Maternal Race**					
White	Reference	Reference	Reference	Reference	Reference
Black or African-American	-187.36*** (-230.53, -144.18)	4.73*** (2.43, 7.04)	0.89 (-5.97, 7.75)	-0.15 (-0.43, 0.12)	2.42 (-1.45, 6.29)
**Maternal Education**					
<9th Grade	Reference	Reference	Reference	Reference	Reference
9th through 11th Grade	-45.32 (-115.60, 24.95)	2.95 (-0.80, 6.70)	-3.32 (-14.49, 7.85)	-0.48 (-1.05, 0.09)	5.85 (-2.20, 13.91)
High School Diploma or GED	-2.88 (-72.96, 67.19)	0.76 (-2.98, 4.50)	-3.35	-0.32 (-0.88, 0.25)	2.37 (-5.61, 10.35)

©2021 Himmelstein G et al. *JAMA Pediatrics*.

			(-14.49, 7.78)		
Some College or Higher	31.71 (-39.58, 103.00)	0.26 (-3.55, 4.06)	-2.72 (-14.05, 8.60)	-0.31 (-0.88, 0.27)	2.28 (-5.75, 10.32)
**Marital Status**					
Unmarried	Reference	Reference	Reference	Reference	Reference
Married	39.28*** (9.50, 69.06)	-0.77 (-2.36, 0.82)	-4.82** (-9.55, -0.09)	-0.06 (-0.24, 0.12)	-0.32 (-2.80, 2.16)

*p<0.1; **p<0.05; ***p<0.01
Ordinary least squares models with zip code and year fixed effects
GED= General Education Diploma

©2021 Himmelstein G et al. *JAMA Pediatrics*.
